# Association of G20210A Prothrombin Gene Mutation and Cerebral Ischemic Stroke in Young Patients

**DOI:** 10.7759/cureus.11984

**Published:** 2020-12-08

**Authors:** Sujan Poudel, Mehwish Zeb, Varshitha Kondapaneni, Sai Dheeraj Gutlapalli, Jinal Choudhari, Olusegun T Sodiya, Ijeoma A Toulassi, Ivan Cancarevic

**Affiliations:** 1 Internal Medicine, California Institute of Behavioral Neurosciences & Psychology, Fairfield, USA; 2 Internal Medicine, Pediatrics, California Institute of Behavioral Neurosciences & Psychology, Fairfield, USA; 3 Pediatrics, Khyber Teaching Hospital, Peshawar, PAK; 4 Orthopedics, California Institute of Behavioral Neurosciences & Psychology, Fairfield, USA; 5 Psychiatry and Behavioral Sciences, California Institute of Behavioral Neurosciences & Psychology, Fairfield, USA; 6 Pathology, California Institute of Behavioral Neurosciences & Psychology, Fairfield, USA

**Keywords:** ‘g20210a prothrombin’ and/or ‘ischemic stroke’

## Abstract

Ischemic stroke is an acute episode of neurological dysfunction resulting from the focal brain and spinal cord infarction. Many etiologies have been reported and vary significantly with the age of the patients. This study aims to show the association of G20210A prothrombin gene mutation and cerebral ischemic stroke in young patients. The prothrombin gene mutation is the second most common inherited thrombophilia after the factor V mutation. In this single missense mutation, guanine is substituted by adenine base pair in the nucleotide position 20210 of the 3'-untranslated region of the prothrombin gene, resulting in abnormal thrombin production predisposing to both arterial or venous thrombosis. Forty-seven relevant articles were selected after a thorough screening process using a regular keyword ‘G20210A Prothrombin’ and/or ‘Ischemic Stroke’ mostly from the PubMed database. We included the studies that are published in the last 22 years with patients age ≤57 years. This review article depicts the association of G20210A prothrombin gene mutation with ischemic stroke in young patients irrespective of ethnicity and zygosity status of their genotype. However, more multicenter prospective studies are needed to better understand the application of prothrombin gene mutation in predicting the associated risk of ischemic stroke in young patients and its importance in deciding the patients' treatment or prognosis.

## Introduction and background

The disease burden of stroke has increased significantly over the last three decades, and now it is the second most common cause of death and disability worldwide [1]. Stroke refers to as a sudden onset of focal neurological deficit attributable to a cause that is vascular in origin [2,3]. It is broadly classified as an ischemic or hemorrhagic stroke. Ischemic stroke is the most common subtype, accounting for 85% of all stroke cases [4]. The global prevalence of stroke is 104.2 million, and that of ischemic stroke is 82.4 million, while the global death from ischemic stroke is 2.7 million [5]. If we look at the United States Centers for Disease Control and Prevention (CDC) data, one in every six deaths from cardiovascular disease was due to stroke; every year, 795,000 people will encounter a stroke and one will occur every 40 seconds [4].

Ischemic stroke is an acute episode of neurological dysfunction resulting from the focal cerebral, spinal, or retinal infarction [2]. The incidence and etiology vary significantly with the age of a patient. Although ischemic stroke is not common in the young population, 10-15% of all ischemic stroke events occur in patients 18-50 years of age [6]. Globally, the annual incidence of ischemic stroke is 1.3 million in adults <50 years of age [7]. Recent studies have suggested that ischemic stroke in younger patients increases along with an increase in traditional risk factors commonly associated with stroke in older adults [6]. Evidence from earlier studies has shown that inherited thrombophilia, including a prothrombin gene mutation, may play a role in this subset of ischemic stroke patients [8,9]. A prothrombin gene mutation is the second most common inherited thrombophilia after factor V Leiden [6]. Typically, a normal prothrombin gene encodes a prothrombin protein, which has a vital role in forming a clot. When the prothrombin mutation is present, commonly a single mutation in the 3'-untranslated region of the prothrombin gene with G-to-A substitution, the body produces an excessive amount of thrombin, leading to the clinical manifestation of either arterial or venous thrombosis [10].

Prothrombin gene mutation in young patients with stroke has been studied widely since its discovery in 1996 [10]. A meta-analysis study in 2014 concluded that ischemic stroke in young patients was associated with prothrombin gene mutation [9]. On the other hand, a case-control study from 2017 stated that prothrombin G20210A mutation has a role only in cerebral sinus venous thrombosis but not in arterial ischemic stroke [11]. Prothrombin gene mutation in ischemic stroke is still unclear due to different studies with contradicting results. This review article aims to show the association between the G20210A prothrombin mutation and stroke in young patients by comparing the results of different relevant studies. It showcases the association of homozygous vs. heterozygous prothrombin gene mutation and ischemic stroke in young patients based on these studies [12-16]. This article further illustrates the ischemic stroke data taking ethnic variation into consideration [17-23].

Forty-seven relevant articles were selected after a thorough screening process using a regular keyword ‘G20210A Prothrombin’ and/or ‘Ischemic Stroke’ mainly from the PubMed database; a few articles were taken from the other sources. We included the studies published in the last 22 years, with subjects age ≤57 years and written in the English language.

## Review

Overview of prothrombin G20210A mutation

**Figure 1:**
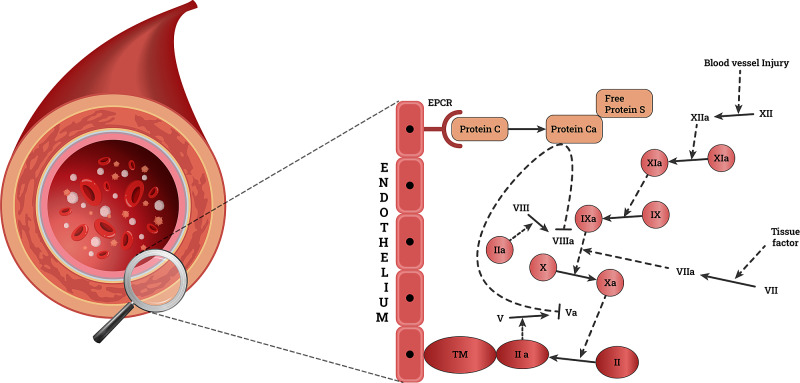
Prothrombin gene mutation increases the thrombin production leading to a hypercoagulability state. a- Activated, EPCR- Endothelium Protein C Receptor, TM- Thrombomodulin, II- Prothrombin, V- labile Factor, VII- Stable Factor, VIII-Antihemophilic Factor, IX- Christmas Factor, X-Stuart-Prower Factor, XI- Plasma Thromboplastin Antecedent and XII- Hageman Factor. Adapted from: step1.medbullets.com/hematology/109066/prothrombin-gene-mutation; www.dreamstime.com/artery-vein-structure-comparison-concept-as-human-circulation-section-blood-vessels-anatomy-close-up-d-illustration-image113607632?fbclid=IwAR01uZBxzajXcCoRMMzXBWPKJpg6D8g9tcrJmZhgdKgXMHsVbk_0zCrZkE8

**Figure 2:**
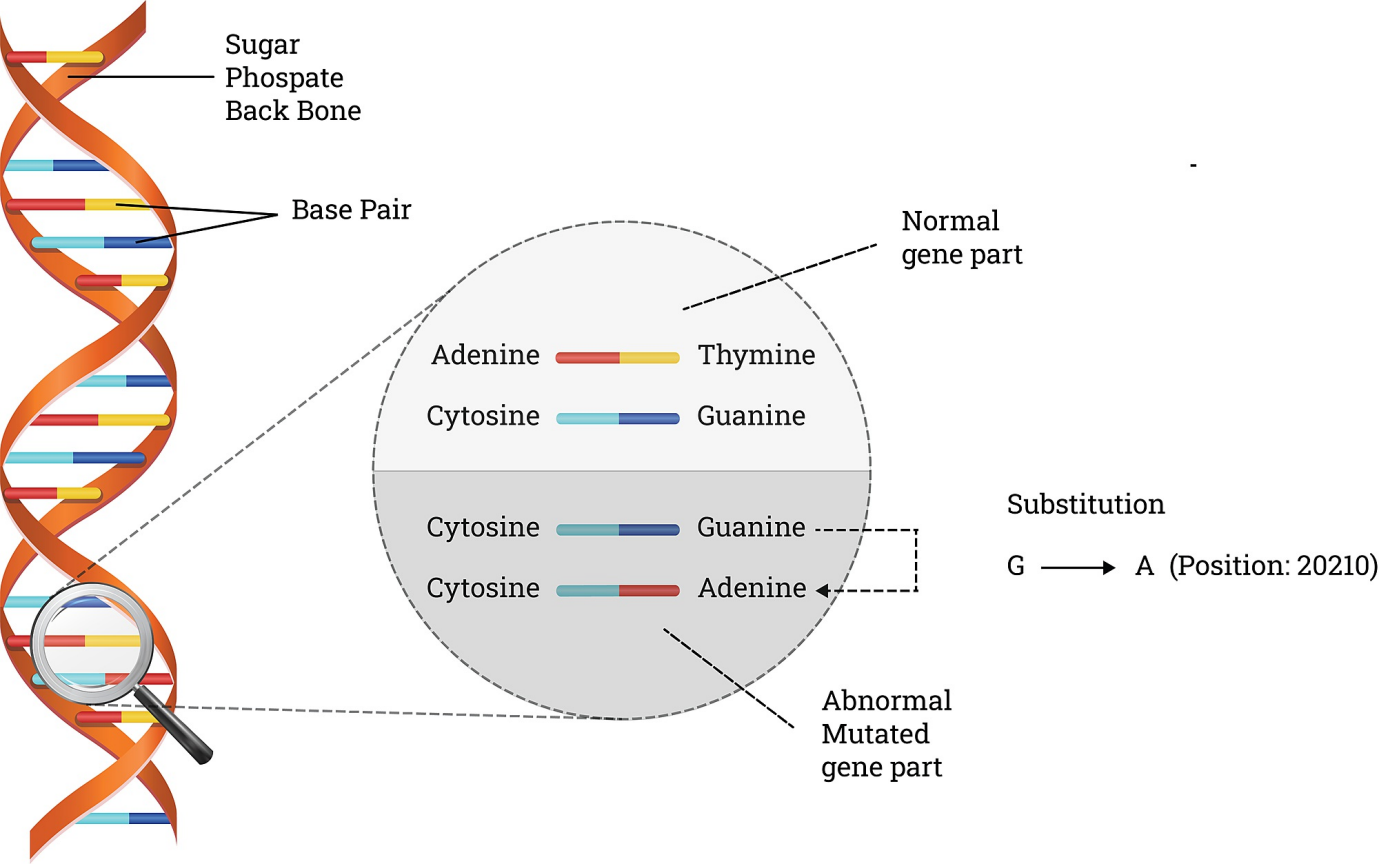
Prothrombin G20210A Mutation G- Guanine & A- Adenine Adapted from: www.coagulationconversation.com/medical/risk-factors-thrombophilia-prothrombin-20210-mutation

Human prothrombin is a vitamin K-dependent glycoprotein synthesized by the liver. It is changed to thrombin by activated factor X (Stuart-Prower factor), which has a vital role in forming the fibrin clot to stop bleeding at the injured site (Figure 1). A prothrombin gene mutation is the second most common mutation after factor V. In patients with prothrombin gene mutation, the guanine is substituted by adenine base pair in nucleotide position 20210 at the 3' untranslated region of the prothrombin gene [10] (Figure 2). This mutation results in unchecked thrombin formation predisposing to the thromboembolic phenomenon. Homozygous and heterozygous are the two different genotypes associated with the prothrombin gene mutation. When the mutation is inherited from both parents, it is said to be homozygous for the gene mutation. If either of the paternal or maternal copies of the prothrombin gene is mutated, it is heterozygous for the gene mutation. Geographical and ethnic differences in carrier frequencies of the prothrombin G20210A mutation appeared between healthy populations with a trend towards lower frequencies from south to north of Europe (range approximately from 0 to 4%) [24]. On the other hand, prothrombin G20210A mutation was found to be very rare or even absent in Asian and African populations and native populations of America (Amerindians) and Australia. In North America, the prevalence ranged from 1.6 to 2.4%, whereas in South America (Brazil), a prevalence of 0.7% was found. The prothrombin mutation seems to increase the risk of arterial thrombosis combined with other established risk factors (e.g. smoking, metabolic risk factors) at a young age. Screening for the presence of the mutation should be performed, at least in cases of otherwise unexplained thrombotic events in adults, as well as in all thrombotic events during childhood, adolescents, or young adults [25]. Direct variant analysis for the prothrombin G20210A allele in patients with clinically suspected thrombophilia using a whole blood specimen may be beneficial for the diagnosis [19-22,26]. This test is a direct alteration analysis of patient blood leukocyte genomic DNA. However, this test has not been cleared or approved by the US Food and Drug Administration [19-22,26]. 

A prothrombin gene mutation is the second most common congenital thrombophilias and considered to be associated with arterial or venous thrombosis risk. Heterozygous and homozygous mutations are the two possible genotypic presentations. If we look at the prevalence data, it is observed that it is more commonly associated with the Caucasian of south European descent, and found less frequently in Asian, African American, Native American, Brazilian and Australian [24,25]. Screening of young patients of otherwise unexplained thrombotic events has been suggested in the past. Still, the direct mutation analysis test has not been approved by the US Food and Drug Administration yet [19-22,26].

The genotype of prothrombin gene mutation and ischemic stroke

Homozygous and heterozygous are the two different forms of genotype associated with the prothrombin gene mutation. Various studies were done in the past, which shows the association of homozygous and heterozygous mutation on ischemic stroke in young patients. In the case-control study done by De Stefano et al. (1998) on 72 patients with a known history of ischemic stroke before the age of 50 years and without any traditional risk factors like hypertension (HTN), diabetes, and hyperlipidemia, it was observed that the mutant prothrombin gene allele frequency (7.6%, 95% confidence interval [CI], 3.3 to 11.9) was significantly higher in cases than in the control group (1.2%; 95% CI, 0.1 to 2.3; P = .0001). The odds ratio (OR) associated with the carriership of the prothrombin gene mutant allele for ischemic stroke in both genotypes was 5.1 (95% CI, 1.6 to 16.3) [27]. According to the Hardy-Weinberg equilibrium, heterozygous genotype was associated with a 3.8-fold increased risk (95% CI, 1.1 to 13.1). On the other hand, assuming an expected prevalence of homozygotes in the general population of 1.6 to 10000, associated with a 208-fold increased risk for cerebral ischemia [27]. A systematic review and meta-analysis done by Chiasakul et al. (2019) assessed prothrombin gene mutation in 45 studies, of which 39 studies were reported to have heterozygosity and homozygosity status. Prothrombin gene mutation was significantly found more in arterial ischemic stroke cases than control irrespective of the patients' zygosity status, with a pooled OR of 1.48 (95% CI, 1.22-1.80). The pooled OR was 0.31 (95% CI, 0.11-0.83; I2=35%) for homozygous prothrombin gene mutation. Of 39 studies tested for the mutation, 31 (79%) did not find homozygous prothrombin gene mutation in either cases or controls. When such studies with zero events were not included in the analysis, the pooled OR for homozygous prothrombin gene mutation was 7.19 (95% CI, 2.47-20.94). For heterozygous prothrombin gene mutation, the pooled OR was 1.41 (95% CI, 1.13-1.76; I2=0%) [28]. Sarecka-Hujar et al. did a meta-analysis study in 2016 that has shown that carriers (heterozygous) of the 20210A allele are significantly present in both children and young adults with arterial ischemic stroke as compared to the control group (P = 0.006; OR, 1.83; 95% CI, 1.19 to 2.80 and P = 0.001; OR, 1.69; 95% CI, 1.25 to 2.28, respectively) [29]. A case report published by Arkel et al. described a patient with ischemic stroke at the age of 22 years and associated inheritance of heterozygous genotype of prothrombin gene 20210A and type I protein C deficiency. They suggested the prothrombin mutation as a risk factor for ischemic stroke [30]. Another case report published by Giardano et al. reported 31 years of women with two episodes of transient ischemic attack and one episode of ischemic stroke and was associated with homozygosity for the 20210A genotype. It was concluded that prothrombin gene mutation has a significant role in arterial and venous thrombosis [31].

The case-control study done by De Stefano et al. showed a significantly increased frequency of mutant prothrombin gene alleles in the patients compared to the control group. Also, the homozygous genotype is associated with a high risk of ischemic stroke in young patients than its heterozygous counterpart [27]. Systematic review and meta-analysis done by Chiasakul et al. stated that irrespective of the zygosity status of the patient, ischemic stroke in a young patient is associated with the prothrombin gene mutation; however, although homozygous prothrombin gene mutation is less frequently found in the studies, exclusion of the study with the zero analysis brought the pooled OR of the homozygous prothrombin gene mutation way higher than its heterozygous counterpart [28]. Another meta-analysis by Sarecka-Hujar et al. showed that carriers of the mutant alleles of the prothrombin gene were found in patients with ischemic stroke at a young age [29]. The two case-control studies by Arkel et al. and Giardano et al. have concluded that ischemic strokes in young patients are associated with heterozygous and homozygous genotypes [30,31]. By comparing and contrasting the above five studies, it is observed that prothrombin gene mutation is seen in young patients with ischemic stroke irrespective of the genotype's zygosity status. However, De Stefano et al. studied that homozygous prothrombin gene mutation is associated with a higher risk of ischemic stroke in young patients [27]. However, the other four studies failed to clearly show a more homozygous association than the heterozygous counterpart of prothrombin gene mutation [28-31].

Prothrombin gene mutation and ethnic variation in ischemic stroke

Studies were done in the past in different geographical regions, including Asia, Europe, Africa, and America, on patients with ischemic stroke at a young age to find an association with the prothrombin mutation. Variations in the results were seen in those studies. In a case-control study done by Saadatnia et al. on investigating the impact of prothrombin gene mutation on the incidence of ischemic stroke in Iranian youth, 76 people of 18 to 50 years of age without the classical risk factors for the stroke were taken for the study (22 cases and 54 controls) and recruited for 26 months in Al-Zahra hospital in Iran. Prothrombin was not observed in any of the patients under study; however, one of the control group samples presented with the heterozygous mutation. They concluded that the prothrombin gene mutation is not considered an independent risk factor for creating an ischemic stroke in young patients [23]. Lichy et al. performed a study in the same region of Germany to investigate the role of persistent foramen ovale (PFO) in causing frequent stroke in younger patients in association with the prothrombin G20210A mutation in the study patients. Patients were assigned into three groups, Group 1 (220 patients) with cerebral ischemia, PFO, and no other etiology, Group 2 (196 patients) with cerebral ischemia of any etiology other than PFO, and Group 3 (362 subjects) with healthy subjects. It was seen from the study that prothrombin G20210A mutation is the strongest risk factor for deep vein thrombosis to be attributing to the stroke risk in a patient with PFO [32]. According to the study done by Pongracz et al. on analyzing the genetic polymorphism as a risk factor for stroke in a cohort of young and elderly stroke patients in Hungary, the prothrombin gene variant was also found to increase in frequency (2.9% European and 4.8% in Hungary) [12]. In young stroke patients (age <50) compared with control subjects, the ORs were higher: in the prothrombin gene (OR: 4.9). Prothrombin G20210A variant was found to be clustered with other prothrombotic genes and described in young stroke victims [12]. A case-control study was done by They-They et al. to explore the relationship between ischemic stroke risk and genetic polymorphism in Casablanca, Morocco. Ninety-one stroke patients were matched with 182 healthy controls, and prothrombin G20210A mutation was assessed. It was observed that the association between prothrombin gene mutation with stroke was insignificant (P = 0.54) [15]. However, if we look at the stroke in large artery disease subtypes, a significant association exists between prothrombin G20210A mutation and ischemic stroke (P = 0.046) [29]. So, it was concluded that prothrombin mutation could be considered a modest risk factor for large artery disease stroke subtypes in the Moroccan population [15]. Salomi et al. conducted a case-control study to find the prevalence of G20210A polymorphism and its association with arterial and venous stroke in both south and north Indians [16]. It was observed that polymorphism is more prevalent in north Indians (5/154; 3.2%) as compared to south Indian (4/516; 0.8%; P = 0.026). Thrombosis was also observed more in north Indian patients (4/82; 4.9%) with ischemic stroke as compared with south Indian patients (1/72, 1.4%; P = 0.003) [30]. It was concluded that PT G20210A is prevalent in India, especially in the North Indian population [16]. A case-control study by Longstreth Jr et al. in western Washington state assessed the risk of ischemic stroke in women with prothrombin gene mutation. Cases were taken from 18 to 44 years of age with a first stroke (n=106), and controls were randomly assigned through random-digit telephone dialing (n=391). The prothrombin variant was observed in 1.9% of cases, one with venous stroke and one with ischemic stroke, and in 1.6% of control subjects. The OR for any stroke was 1.48 (95% CI, 0.14 to 9.17). ORs for stroke types were also not statistically significant. It was concluded that the prothrombin gene variant is not an important risk factor for stroke in young patients [33]. A case-control study was done by Chang et al. to explore the association of prothrombin G20210A mutation with arterial ischemic stroke in 49 patients with thrombosis and 46 healthy Chinese persons. Prothrombin G20210A variant was not seen in both the patient and control groups, and the conclusion was drawn that this mutation may not be a major risk factor for thrombogenesis in Chinese people [34].

Seven different studies were analyzed to determine the association of the prothrombin mutation in young patients with ischemic stroke in different geographical areas. In the study in Iranian youth, it was observed that prothrombin gene mutation was not associated with ischemic stroke in patients 18 to 50 years old without any traditional risk factors for the stroke [23]. One of Germany's studies showed that PFO was a significant risk factor for developing ischemic stroke in young patients associated with prothrombin G20210A Mutation [32]. Studies done in Hungary showed that prothrombin gene mutation clustering with other prothrombotic genes was associated with ischemic stroke in young [12]. A study was done in Casablanca, Morocco, that concluded prothrombin gene mutation is associated with moderate risk for ischemic stroke in large artery disease subtypes of stroke [15]. If we look at the data of a study done in India, it was found that prothrombin mutation is present more in northern Indian patients than south Indians [16]. A study done in Washington state of the United States has shown that prothrombin gene mutation is not a significant risk factor for stroke in young women of the western part of the state [33]. Chang et al.'s study in the Chinese population suggested that the prothrombin gene variant is not present in the cases and controls within the study population and may not be a significant risk for arterial thrombosis in Chinese people [34]. Overall, the prothrombin gene mutation is associated with ischemic stroke cases in young patients in most of the above studies. Still, the variation observed in different geographical regions cannot be clearly explained.

Fundamental studies that show the association between prothrombin G20210A mutation and ischemic stroke in young patients

Table 1 provides an overview of various studies showing the association between prothrombin gene mutation and ischemic stroke in young patients.

**Table 1:** Overview of the fundamental studies that show the association between prothrombin gene mutation and ischemic stroke in young patients. OR-Odds Ratio, CI- Confident Interval, FII- Factor II, FV- Factor V, PT-Prothrombin, PFO- Patent Foramen Ovale

reference Country/Year	Age Group	Study Design	Study Aim	Sample size	Results
Arkel YS et al. USA/1998 [30]	22years	Case report	To determine that the reported cases of ischemic stroke and protein c deficiency may have had other prothrombotic disorders such as the prothrombin mutation.	Single-subject	To study all patients with premature stroke for prothrombin mutation and the other risk factors for thrombosis.
V De Stefano et al. Italy/1998 [27]	<50 years of age	Case-control study	To determine whether the Prothrombin G20210A mutant genotype is a risk factor for ischemic cerebrovascular disease in young patients.	Seventy-two patients (35 male and 37 female) were diagnosed with ischemic stroke, and without any risk factors, 198 thrombosis-free individuals were in the control group.	The mutant factor II allele frequency in the patient group (7.6%, 95% confidence interval [CI], 3.3 to 11.9) was significantly higher than in the controls (1.2%; 95% CI, 0.1 to 2.3; P = .0001). The odds ratio for ischemic stroke associated with the carriership of the mutant factor II allele (both heterozygous and homozygous genotypes) was 5.1 (95% CI, 1.6 to 16.3)
G Young et al. USA/2003 [8]	<18 years	Consortium study	To determine the clinical manifestations of the prothrombin mutation in children.	38 children	37% (14 out of 38) of children had central nervous system thrombosis
Pezzini A et al. Italy/2003 [35]	<45 years	Case-control study	To investigate the association between inherited thrombophilic disorders and PFO-related strokes in a series of young adults in the setting of a case-control study.	125 consecutive subjects (age, 34.7±7.3 years) with ischemic stroke and 149 age- and sex-matched control subjects	The PT(G20210A) variant was more frequent in the PFO+ group compared with control subjects and the PFO- group (PFO+ versus control subjects, 11% versus 2%; 95% CI, 0.04 to 0.94; PFO+ versus PFO-, 11% versus 1.1%; 95% CI, 1.09 to 109; P=0.047).
Justo Aznar et al. Spain/2004 [36]	<50 years	Case-control study	To determine the role of Factor V Leiden and prothrombin G20210A mutations in young adults with cryptogenic ischemic stroke.	49 patients with cryptogenic stroke and compared with controls	The odds ratio (OR) for cryptogenic stroke was 3.75 (95% CI, 1.05-13.34) for PT 20210.
Kim RJ et al. USA/2003 [37]	<55years Subgroup analysis	Meta-analysis	To determine the association between factor V Leiden, prothrombin G20210A, and methylenetetrahydrofolate reductase C677T mutations and events of the arterial circulatory system.	A total of 56 studies and 54,547 persons served as a basis of the final analysis	The association between G20210A mutation and the arterial ischemic event was modest (OR, 1.32; 95% CI, 1.03-1.69) Subgroup analyses of younger patients revealed a slightly stronger association overall.
Jiang B et al. USA/2014 [9]	<55 years	Meta-analysis	To determine the association between prothrombin G20210A and ischemic stroke in a white case-control population followed by a metanalysis of the studies.	2305 cases of European ancestry white population	Prothrombin mutation was associated with significantly increased stroke risk in adults ≤55 years (OR=1.4; 95% CI=1.1-1.9; P=0.02), with significance increasing with the addition of the GEOS results (OR=1.5; 95% CI=1.1-2.0; P=0.005)
Sarecka-Hujar B et al. Poland/2017 [29]	Children, Young adults	Meta-analysis	To address the relation between the FII 20210G>A polymorphism and arterial ischemic stroke.	3586 Cases and 6440 Control Subjects	Carriers of 20210A allele of the prothrombin gene are commonly seen in patients with arterial ischemic stroke, including children and young adults, as compared to the control group (P = 0.006; odds ratio, 1.83; 95% confidence interval, 1.19 to 2.80 and P = 0.001; odds ratio, 1.69; 95% confidence interval, 1.25 to 2.28, respectively)
Thita Chiasakul et al. Thailand, USA, Canada/2019 [28]	Adult patients Age>15 years	Meta-analysis	To evaluate the association of inherited thrombophilia and the risk of arterial ischemic stroke in adults.	Sixty-eight eligible studies enrolled 11916 stroke patients and 96057 controls.	As compared to the control group, patients with arterial ischemic stroke were significantly more likely to have the inherited thrombophilias, including prothrombin G20210A mutation (OR, 1.48; 95% CI, 1.22–1.80; I^2^=0%).
Solomi BSB et al. India/2019 [16]	Mean age 36.7 years (cases) mean age 37.6 years (controls)	Case-control study	To determine the prevalence and association of the prothrombin G20210A polymorphism in patients with arterial and venous strokes.	82 patients from north India and 310 patients from south India 278 healthy, age- and sex-matched controls	The heterozygous allele of the polymorphism was detected in both groups with significantly higher prevalence among North Indians (5/154; 3.2%) compared with south Indians (4/516; 0.8%; p = 0.026).Thrombosis as a manifestation of this polymorphism was more among north Indians, with 4/82 (4.9%) of patients with ischemic stroke and cerebral venous thrombosis having this polymorphism compared with South Indian patients 1/72 (1.4%), p = 0.003.

## Conclusions

This review article shows the association of G20210A prothrombin gene mutation with ischemic stroke in young patients irrespective of ethnicity and zygosity status of their genotype. The homozygous genotype of prothrombin gene mutation presents as a more significant risk factor for arterial thrombosis; however, the heterozygous genotype is more commonly seen in patients with ischemic stroke. Variation is observed in the prevalence of prothrombin gene mutation in stroke patients of different ethnicities, but its occurrence is still unclear. Most prothrombin gene mutations related to stroke are present in patients ≤57 years of age. Despite the better understanding of prothrombin G20210A mutation, this gene analysis's importance in deciding the patients' treatment or prognosis is not commonly practiced in the clinical ground. More multicenter prospective studies are needed in the future to know better about the usefulness of prothrombin gene mutation in predicting the associated risk for ischemic stroke in young patients.

## References

[1] Feigin V, Norrving B, Sudlow CM, Sacco RL (2018). Updated criteria for population-based stroke and transient ischemic attack incidence studies for the 21st century. Stroke.

[2] Sacco RL, Kasner SE, Broderick JP (2013). An updated definition of stroke for the 21st century: a statement for healthcare professionals from the American Heart Association/American Stroke Association. Stroke.

[3] French BR, Boddepalli RS, Govindarajan R (2016). Acute ischemic stroke: current status and future directions. Mo Med.

[4] Mozzafarian D, Benjamin EJ, Go AS (2016). Heart disease and stroke statistics-2016 update: a report from the American Heart Association. Circulation.

[5] Virani SS, Alonso A, Benjamin EJ (2020). Heart disease and stroke statistics-2020 update: a report from the American Heart Association. Circulation.

[6] George MG (2020). Risk factors for ischemic stroke in younger adults: a focused update. Stroke.

[7] Putaala J, Martinez-Majander N, Saeed S (2017). Searching for Explanations for Cryptogenic Stroke in the Young: Revealing the Triggers, Causes, and Outcome (SECRETO): rationale and design. Eur Stroke J.

[8] Young G, Manco-Johnson M, Gill JC (2003). Clinical manifestations of the prothrombin G20210A mutation in children: a pediatric coagulation consortium study. J Thromb Haemost.

[9] Jiang B, Ryan KA, Hamedani A (2014). Prothrombin G20210A mutation is associated with young-onset stroke: the genetics of early-onset stroke study and meta-analysis. Stroke.

[10] Varga EA, Moll S (2004). Prothrombin 20210 mutation (Factor II Mutation). Circulation.

[11] Beye A, Pindur G (2017). Clinical significance of factor V Leiden and prothrombin G20210A-mutations in cerebral venous thrombosis - comparison with arterial ischemic stroke. Clin Hemorheol Microcirc.

[12] Pongrácz E, Tordai A, Csornai M (2002). Genetics of blood coagulation in young stroke patients [Article in Hungarian]. Ideggyogy Sz.

[13] Eterović D, Titlić M, Culić V, Zadro R, Primorac D (2007). Lower contribution of factor V Leiden or G202104 mutations to ischemic stroke in patients with clinical risk factors: pair-matched case-control study. Clin Appl Thromb Hemost.

[14] Voetsch B, Damasceno BP, Camargo EC (2000). Inherited thrombophilia as a risk factor for the development of ischemic stroke in young adults. Thromb Haemost.

[15] They-They TP, Battas O, Slassi I, Rafai MA, Katumbay DT, Nadifi S (2012). Prothrombin G20210A and factor V Leiden polymorphisms in stroke. J Mol Neurosci.

[16] Salomi BB, Christudass CS, Aaron S, Turaka VP (2019). Prothrombin G20210A polymorphism in patients with venous and cryptogenic arterial strokes among ethnic groups in south and north India. Natl Med J India.

[17] Howard TE, Marusa M, Boisza J (1998). The prothrombin gene 3'-untranslated region mutation is frequently associated with factor V Leiden in thrombophilic patients and shows ethnic-specific variation in allele frequency. Blood.

[18] Poort SR, Rosendaal FR, Reitsma PH, Bertina RM (1996). A common genetic variation in the 3'untranslated region of the prothrombin gene is associated with elevated plasma prothrombin levels and an increase in venous thrombosis. Blood.

[19] Makris M, Preston FE, Beauchamp NJ (1997). Co-inheritance of the 20210 A allele of the prothrombin gene increases the risk of thrombosis in subjects with familial thrombophilia. Thromb Haemost.

[20] De Stefano V, Martinelli I, Mannucci PM (1999). The risk of recurrent venous thrombosis among heterozygous carriers of both factor V Leiden and the G20210A prothrombin mutation. N Engl J Med.

[21] Hall JG, Eis PS, Law SM (2000). Sensitive detection of DNA polymorphisms by the serial invasive signal amplification reaction. Proc Natl Acad Sci USA.

[22] Heit JA (2007). Thrombophilia: clinical and laboratory assessment and management. Consultative Hemostasis and Thrombosis.

[23] Saadatnia M, Salehi M, Amini G, Seyyed Agha Miri N (2012). The impact of prothrombin (G20210A) gene mutation on stroke in youths. ARYA Atheroscler.

[24] Junker R, Nowak-Göttl U (1998). The prothrombin G20210A mutation- a common cause of thrombophilia?. J Lab Med.

[25] Jadaon MM (2011). Epidemiology of prothrombin G20210A mutation in the Mediterranean region. Mediterr J Hematol Infect Dis.

[26] Poort SR, Rosendaal FR, Reitsma PH, Bertina RM (1996). A common genetic variation in the 3'untranslated region of the prothrombin gene is associated with elevated plasma prothrombin levels and an increase in venous thrombosis. Blood.

[27] De Stefano V, Chiusolo P, Paciaroni K (1998). Prothrombin G20210A mutant genotype is a risk factor for cerebrovascular ischemic disease in young patients. Blood.

[28] Chiasakul T, De Jesus E, Tong J (2019). Inherited thrombophilia and the risk of arterial ischemic stroke: a systematic review and meta-analysis. J Am Heart Assoc.

[29] Sarecka-Hujar B, Kopyta I, Skrzypek M, Sordyl J (2017). Association between the 20210G>A prothrombin gene polymorphism and arterial ischemic stroke in children and young adults-two meta-analyses of 3586 cases and 6440 control subjects in total. Pediatr Neurol.

[30] Arkel YS, Ku DH, Gibson D, Lam X (1998). Ischemic stroke in a young patient with protein C deficiency and prothrombin gene mutation G20210A. Blood Coagul Fibrinolysis.

[31] Giordano P, De Lucia D, Coppola B, Iolascon A (1999). Homozygous prothrombin gene mutation and ischemic cerebrovascular disease: a case report. Acta Haematol.

[32] Lichy C, Reuner KH, Buggle F (2003). Prothrombin G20210A mutation, but not factor V Leiden, is a risk factor in patients with persistent foramen ovale and otherwise unexplained cerebral ischemia. Cerebrovasc Dis.

[33] Longstreth WT, Rosendaal FR, Siscovick DS (1998). Risk of stroke in young women and two prothrombotic mutations: Factor V Leiden and prothrombin gene variant (G20210A). Stroke.

[34] Chang JH, Zhang GS (2000). Frequency of prothrombin gene G20210A variant in the 3'-untranslated region in Chinese people with ischemic stroke [Article in Chinese]. Hunan Yi Ke Da Xue Xue Bao.

[35] Pezzini A, Del Zotto E, Magoni M (2003). Inherited thrombophilic disorders in young adults with ischemic stroke and patent foramen ovale. Stroke.

[36] Aznar J, Mira Y, Vayá A, Corella D, Ferrando F, Villa P, Estellés A (2004). Factor V Leiden and prothrombin G20210A mutations in young adults with cryptogenic ischemic stroke. Thromb Haemost.

[37] Kim RJ, Becker RC (2003). Association between factor V Leiden, prothrombin G20210A, and methylenetetrahydrofolate reductase C677T mutations and events of the arterial circulatory system: a meta-analysis of published studies. Am Heart J.

